# RRx-001, A novel dinitroazetidine radiosensitizer

**DOI:** 10.1007/s10637-016-0326-y

**Published:** 2016-02-03

**Authors:** Bryan Oronsky, Jan Scicinski, Shoucheng Ning, Donna Peehl, Arnold Oronsky, Pedro Cabrales, Mark Bednarski, Susan Knox

**Affiliations:** EpicentRx Inc, 800 W El Camino Real, Suite 180, Mountain View, CA 94040 USA; Stanford University, 875 Blake Wilbur Dr Clinic D, Stanford, CA 94305 USA; InterWest Partners, 2710 Sand Hill Rd #200, Menlo Park, CA 94025 USA; Department of Bioengineering, University of California San Diego (UCSD), 9500 Gilman Dr, La Jolla, CA 92093 USA

**Keywords:** Radiosensitizer, Radiosensitization, Chemosensitizer, Cancer, Clinical trials, RRx-001

## Abstract

The ‘holy grail’ in radiation oncology is to improve the outcome of radiation therapy (RT) with a radiosensitizer—a systemic chemical/biochemical agent that additively or synergistically sensitizes tumor cells to radiation in the absence of significant toxicity. Similar to the oxygen effect, in which DNA bases modified by reactive oxygen species prevent repair of the cellular radiation damage, these compounds in general magnify free radical formation, leading to the permanent “fixation” of the resultant chemical change in the DNA structure. The purpose of this review is to present the origin story of the radiosensitizer, RRx-001, which emerged from the aerospace industry. The activity of RRx-001 as a chemosensitizer in multiple tumor types and disease states including malaria, hemorrhagic shock and sickle cell anemia, are the subject of future reviews.

## Introduction

The origin story of the Phase II anticancer agent RRx-001 is presented in this review.

Unlike the pharmaceutical industry’s emphasis on “me too” drugs that slavishly mimic existing chemical compounds (e.g., statins, antibiotics, H1 and H2 histamine blockers etc.), RRx-001, having been derived from the aerospace and defense sector, is the prototype of a pharmacologically unprecedented and decidedly “non-me too” chemical class called dinitroazetidines.

The strategic decision to move forward a compound hinges on a risk benefit analysis. In this case the development of RRx-001 was predicated on the reasonable anticipation of non-toxicity, even though the benefits initially were unknown. Given that the detonation of nitrogenous combustibles in the atmosphere is a potential threat to the health of humans, livestock, wildlife, and ecosystems, military agencies in the US have conducted risk assessments [1]; in particular, the safety profile of TNAZ [2], structurally similar to RRx-001 had already been comprehensively characterized, suggesting that the development of dinitroazetidine containing compounds were inherently less risky. The availability of toxicology information was a significant advantage since GLP-repeated dose toxicology studies cost millions of US dollars [3] and take up to or over a year to complete.

In addition, since the chemistry of energetic compounds is based on free radical-initiated chain reactions, synergy with radiation therapy was suggested, given that the outcome of the latter depends on the generation of reactive oxygen species [4]. Free radical production was also expected because 1,3,3 trinitroazetidine (TNAZ) [5], an explosive propellant for guns, artillery, mortars and rockets, and the closest chemical analog of RRx-001, yielded free radicals during bond cleavage [6].

The only examples of pharmaceutical agents with comparable origins to RRx-001 are the antituberculant, iproniazid, and the antidepressant imipramine [7], derived from leftover World War II rocket fuel hydralazine as well as the explosive nitroglycerin (NTG), introduced as a treatment for angina pectoris [8] several years before Alfred Nobel, the inventor of dynamite, developed the condition [9]. TNAZ modified with the removal of a single nitro group (NO_2_) and substitution of a bromoacetate group produced a non-explosive derivative called ABDNAZ, an acronym for 1-bromoacetyl-3,3-dinitroazetidine, a name later shortened to RRx-001 for easier-to-use pronunciation and communication. *In vivo* RRx-001 demonstrated single-agent activity as well as hypoxic cell radiosensitization [10].

## Effects of hypoxia on radiosensitivity

When solid tumor growth exceeds a critical diameter of 1–2 mm^3^ (10^6^ cells) [11], diffusion limitations of oxygen and nutrients from blood vessels located in the periphery leads to necrotic centers. The resultant activation of the hypoxia inducible factor (HIF) system mediates the expression of VEGF, erythropoietin and factors regulating glucose transport and glycolysis such as GLUT-1 and GLUT-3, [12]; the induction of these genes drives vascular remodeling and a metabolic switch to aerobic glycolysis, which are integral to malignant transformation and progression. [13, 14] Due to a dearth of superoxide, hydrogen peroxide and hydroxyl radicals that oxidatively damage macromolecules including lipid, protein and nucleic acid under low oxygen conditions, the presence of hypoxia predicts for a poor response to radiotherapy.[15] For example, nearly 40 % of breast cancers have hypoxic regions with oxygen concentrations below the threshold required for half-maximal radiosensitivity (pO2 < 2.5 mmHg), which adversely impacts the response to radiotherapy [16].

As a common feature of most solid tumors, hypoxia, therefore, plays a critical role not only in the development of radioresistance but also chemoresistance. Unlike tumors, and with the exception of tissues like the retina and the dermis, normal cells are normally well-oxygenated (>10 mm/Hg O_2_) [17]. A clear therapeutic disadvantage, hypoxia is also potentially an exploitable physiological difference, opening the door to the development of hypoxia-selective agents that are preferentially toxic only to oxygen-deficient tumor cells. In particular, the development of the nitroimidazoles as hypoxic cell sensitizers that mimic the effect of oxygen on tumors resulted from the discovery that ^14^C-labelled misonidazole bound selectively to macromolecules in hypoxic cells both *in vitro* and in vivo [18] and were reduced by nitroreductase enzymes to a radical anion—this reduction only occurs under hypoxic conditions [19].

## Radiosensitizers defined

The term “radiosensitizer” refers to an agent that enhances the therapeutic ratio of radiotherapy for similar levels of normal tissue toxicity, which is tantamount to the Holy Grail in radiation oncology and cancer therapy in general because selective cytotoxicity predicts improved patient tolerance and overall quality of life. Like the reaction of oxygen, which leads to the formation of DNA hydroxyl and peroxyl radicals that directly attack DNA, radiosensitizers increase the pool of oxidizing species, resulting in enhanced “fixation” of free-radical DNA damage [20].

Unfortunately, however, the history of radiosensitization is associated with the limited clinical efficacy and substantial normal tissue toxicity observed with potential radiosensitizers including the halogenated pyrimidines [21] and other antimetabolites, cisplatin and 5-fluorouracil (5-FU), the nitroimidazoles, and the hypoxic cytotoxins such as tirapazamine and the mitomycin-related quinones EO9 and porfiromycin [22, 23].

The lessons learned from the failure of these compounds is that a radiosensitizer should ideally possess or exhibit:

1. Systemic single agent activity
2. Tumor specificity
3. Sequence-dependent synergy with radiation with no overlapping toxicity
4. Activation under hypoxia
5. Broad therapeutic index
6. Normal tissue radioprotection and tumor radiosensitization

Given its novel redox-based mechanism of radiosensitization, favorable toxicity profile, and inherent cytotoxicity, RRx-001 fits the definition of a promising carcinoma radiosensitizer, based on the criteria listed above.

## RRx-001 radiosensitization properties

### Inherently selective cytotoxicity

**Fig. 1:**
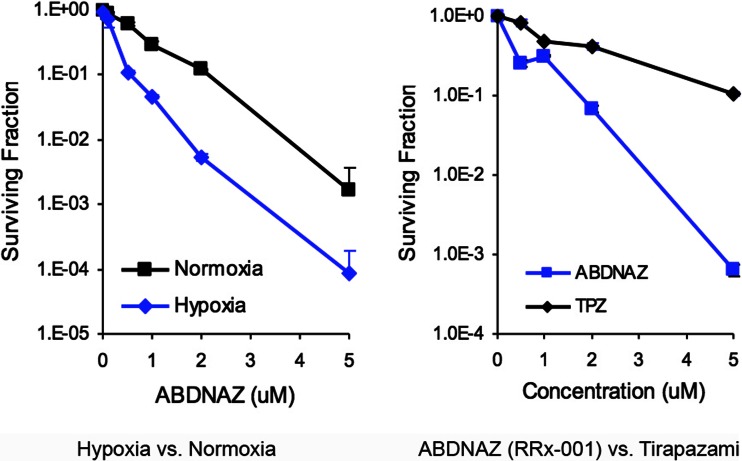
The in vitro activity of RRx-001 (ABDNAZ) under normoxia or hypoxia in SCC VII tumor

RRx-001 is an optimized derivative of TNAZ, a compound chosen from a collection of energetic polynitro propellant materials on the basis of a greater increase in IC50 hypoxia compared to normoxia (Fig. 1) [10].

**Fig. 2:**
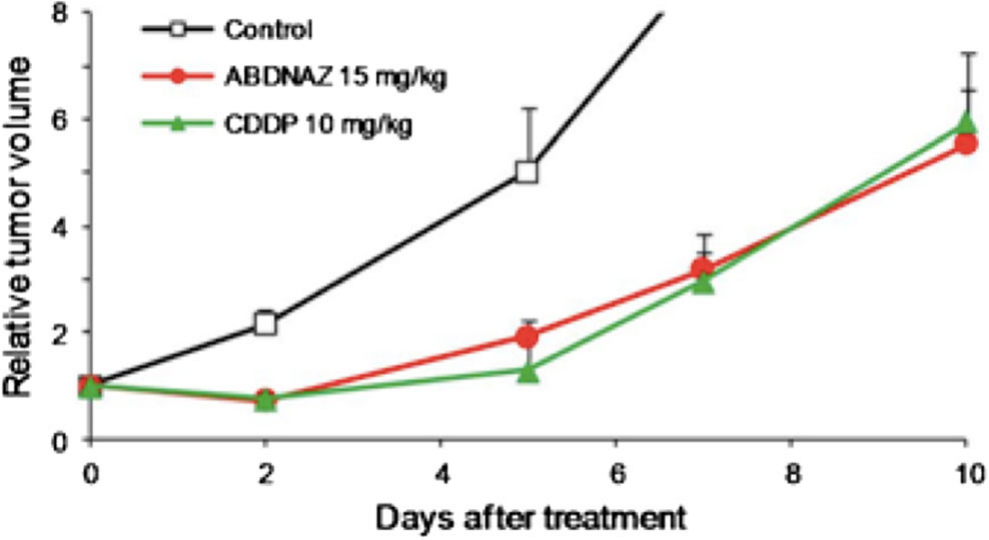
A comparison of the single dose antitumor activity of RRx-001 (ABDNAZ) to cisplatin in murine SCCVII tumor model

*In vivo* activity of RRx-001 in the SCCVII syngeneic mouse tumor model demonstrated equivalent activity to Cisplatin, with no apparent induced side effects, as shown below (Fig. 2), indicative of promising antitumor activity and a favorably low acute toxicity profile [10].

### Synergistic effects with radiation

**Fig. 3:**
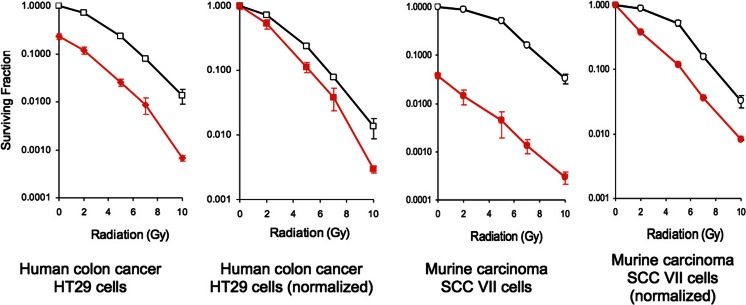
RRx-001 (ABDNAZ) in human colon cancer HT29 and murine carcinoma SCC VII cell lines: radiosensitization effects

**Fig. 4:**
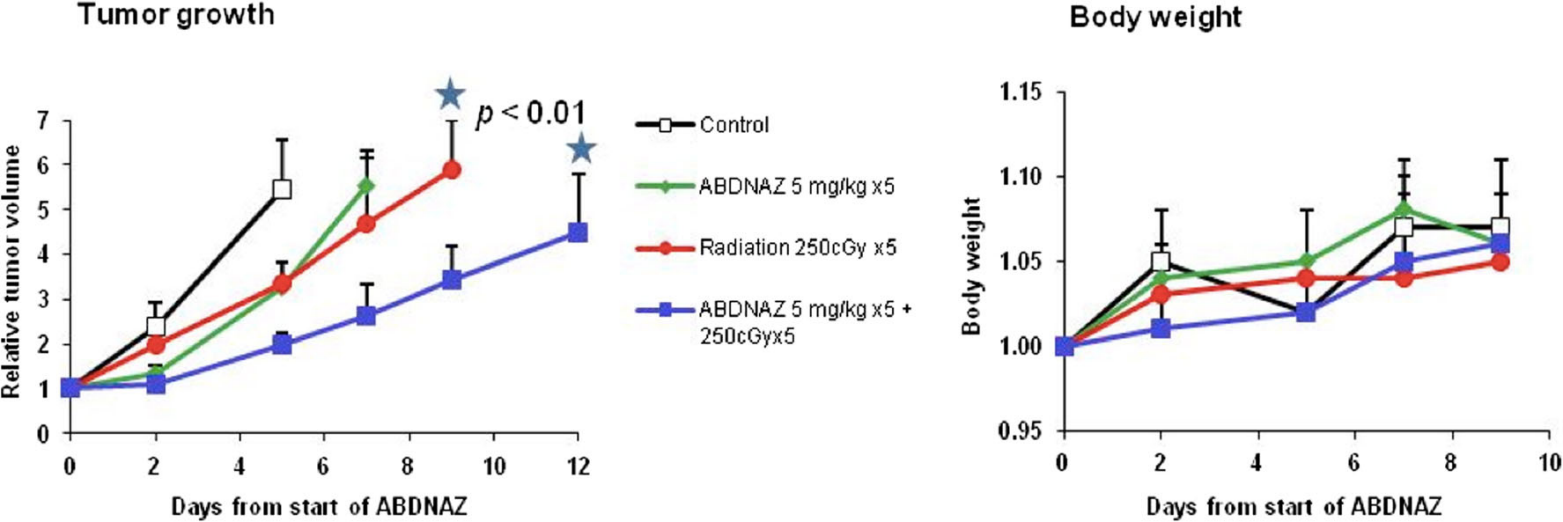
RRx-001 (ABDNAZ) in murine SCCVII tumor model: potentiation of the effect of radiation. RRx-001 (ABDNAZ) was given at a dose of 5 mg/kg QD for 5 days. Radiotherapy: 250 cGy QD for 5 days

*In vitro* experiments showed that RRx-001 synergistically enhances XRT-induced inhibition of proliferation of both radiosensitive SCCVII cells and relatively radioresistant HT-29 tumor cells (Fig. 3), potentiates the survival of SCCVII tumor-bearing mice, and significantly improves the therapeutic ratio of radiotherapy (Fig. 4). Analysis with Jin’s formula (*Q* = *Ea* + *b*/(*Ea* + *Eb* − *Ea* × *Eb*) [24] of both in vitro and in vivo experiments for antagonism, additive effects, and synergism revealed a synergistic interaction between RRx-001 and radiation.

**Fig. 5:**
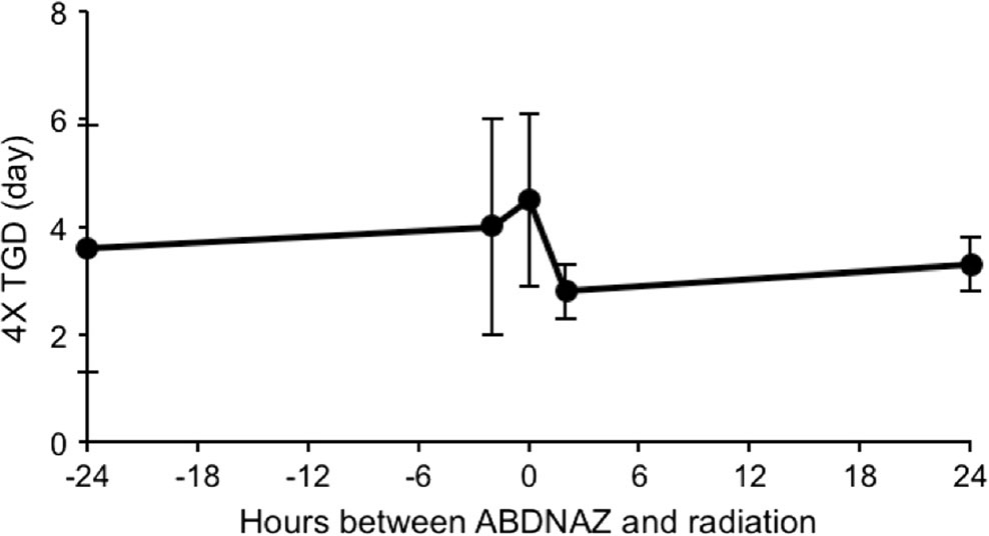
Tumor growth delay (TGD) and time between RRx-001 (ABDNAZ) dosage and radiation in murine SCCVII tumor model. *p* = 0.05 RRx-001 (ABDNAZ) *t* = 0 and *t* = 24 h; *p* = 0.09 RRx-001 (ABDNAZ) *t* = 0 and *t* = 2 h

Potentiation of radiation-induced growth delay in murine tumors was both dose and schedule dependent. Maximum tumor growth delay occurred when RRx-001 was administered minutes prior to or concomitant with (during) radiation (Fig. 5).

The mechanistic basis of radiosensitization is mediated by an intricate interaction of RRx-001-modified RBCs (on administration RRx-001 penetrates the red blood cell membrane and binds irreversibly to a particular residue hemoglobin, beta Cysteine 93) with the tumor vasculature. [25, 26] The preferential adhesion of RRx-001 RBCs to the vascular endothelium is followed by tumor internalization and catabolization in a Trojan Horse manner, releasing redox active RRx-001 and RBC metabolites (i.e. nitric oxide [26, 27] iron and heme) EpicentRx unpublished data.

**Fig. 6:**
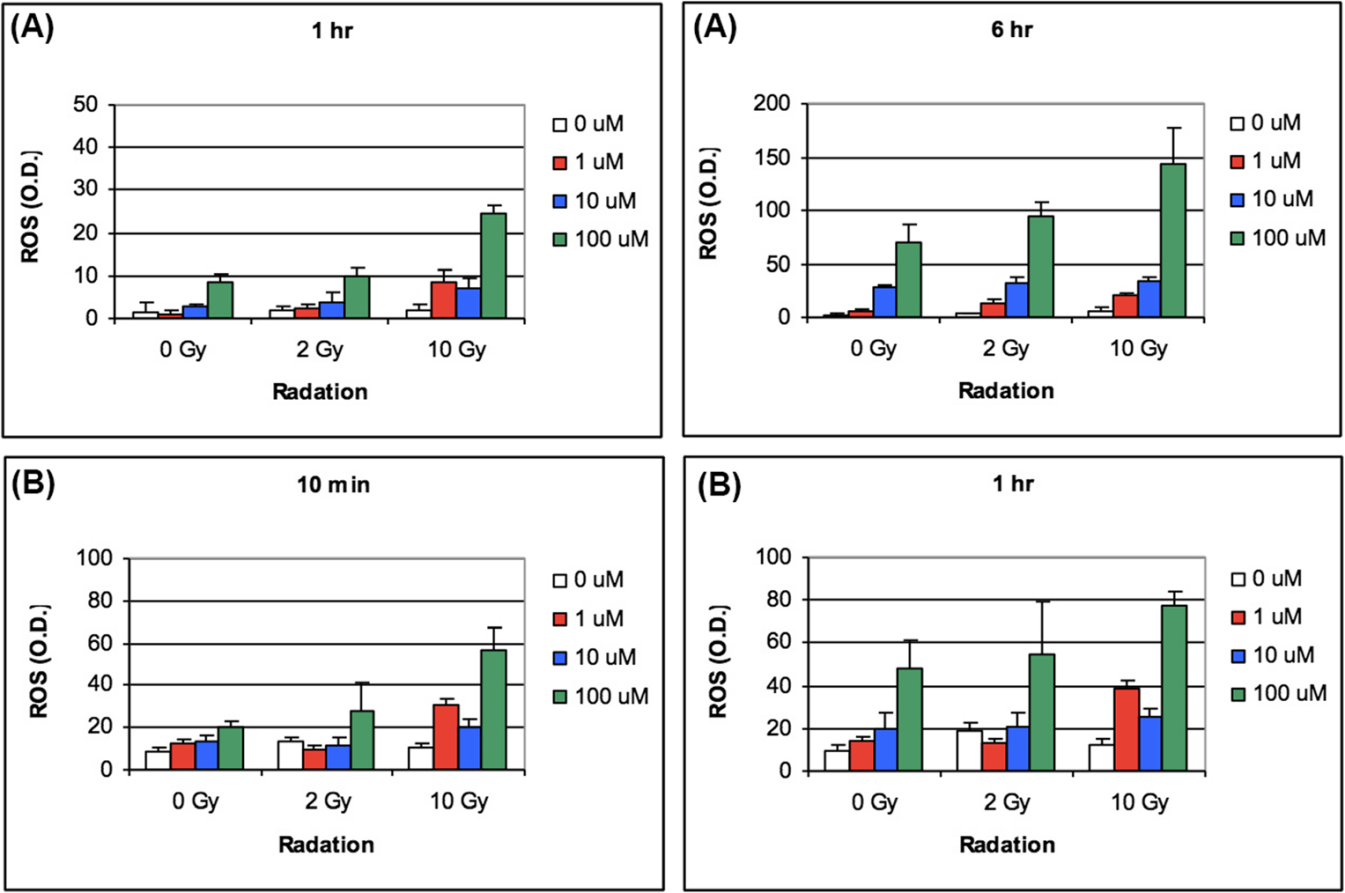
**a** Generation of ROS over time in HT29 tumor cells by RRx-001 (ABDNAZ) with and without radiation. **b** Generation of ROS in SCVII tumor cells by RRx-001 (ABDNAZ) with and without radiation

**Fig. 7:**
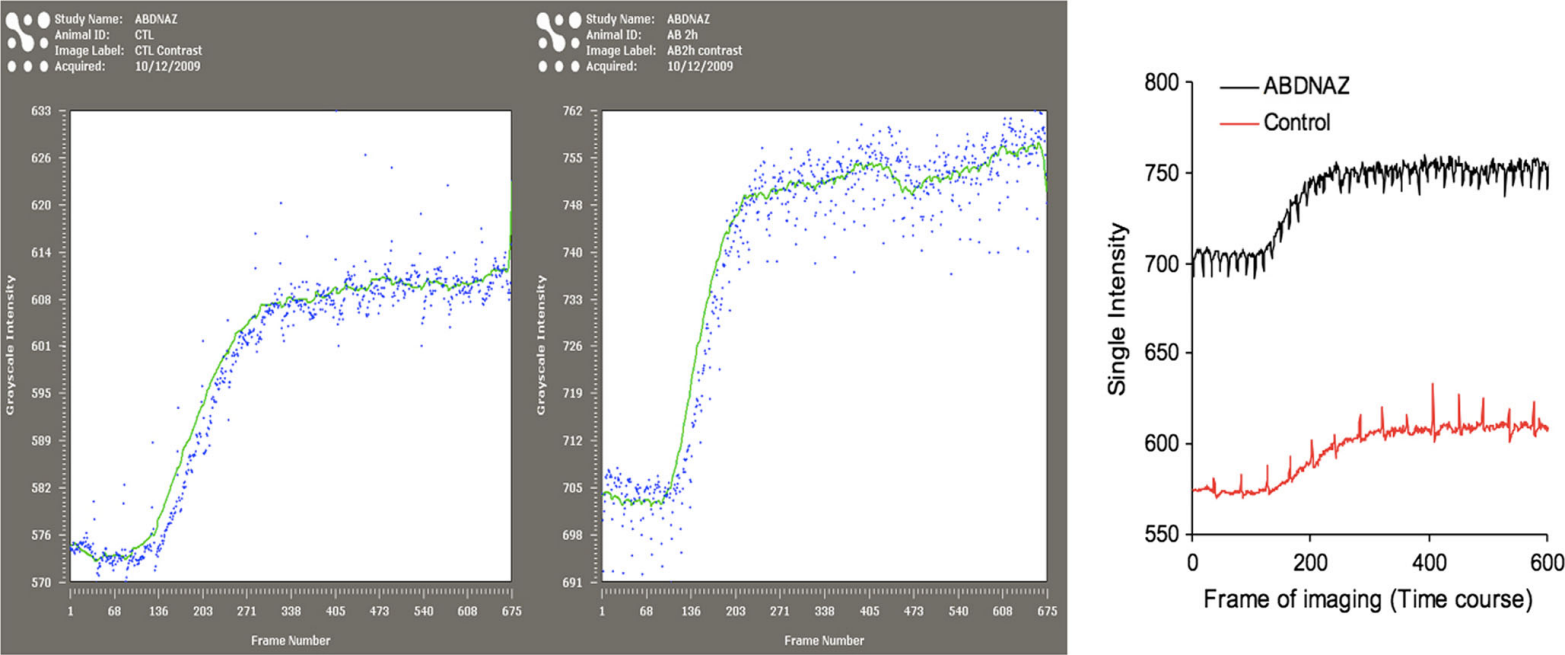
RRx-001 (ABDNAZ) causes and increase in blood perfusion and blood volume in murine SCCVII tumor model. The slope represents relative rate of tumor blood perfusion and the level of the plateau represents relative blood volume

**Fig. 8:**
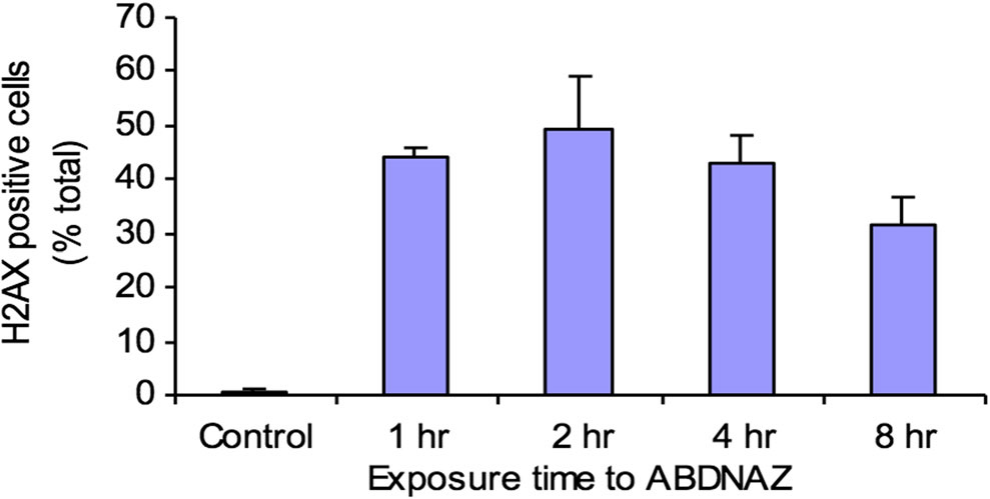
RRx-001 (ABDNAZ) induced DNA damage measured by degree of induction of γH2AX positive cells

The beneficial pleiotropic effects of this oxidative damage include: nitric oxide generation, [28] increased tumor perfusion, cell cycle arrest, apoptosis and inhibition of cell division, inhibition of epigenetic enzymes responsible for DNA methylation and various histone modifications [29] and effects on DNA damage and repair pathways. This underlying mechanism of action is suggested by an expanding body of preclinical evidence: 1) in a dose dependent manner, RRx-001 enhanced radiation-induced pro-oxidant production (Fig. 6). 2) RRx-001 significantly improved tumor blood flow/perfusion from baseline values compared to control in a murine SCCVII tumor model. The enhanced blood flow and, by extension, oxygenation may be, at least, in part, related to the overproduction of nitric oxide (NO) via RRx-001-modified deoxyhemoglobin under hypoxic conditions that are specific to cancer cells (Fig. 7). Exposure of HT-29 cells RRx-001 results in the formation of a dose-dependent increase in DNA double strand breaks assessed by the measurement of gamma-H2AX, a biomarker of DNA damage (Fig. 8) [10].

## Conclusion and future directions

Despite the demonstration of activity as a chemosensitizer, chemo-resensitizer [29, 30] and immunosensitizer in multiple tumor types, RRx-001 has continued along the development path of radiosensitization: currently two Phase I/II clinical trials are underway in brain metastases with whole brain radiotherapy (WBRT) and in primary GBM with radiation and temozolomide. The emerging data is highly positive, albeit limited and preliminary. These caveats notwithstanding, the activity profile of RRx-001 + radiation therapy suggests synergistic cancer cell cytotoxicity in the absence of *any* neurological toxicity, which would likely support multicenter Phase III clinical trials with concurrent radiation and chemoradiation in these indications.

The percentage of cancer patients that will receive radiation therapy (RT) at some time during their course of their disease is approximately 50–60 %.[31] A well-characterized dose-response relationship between malignant and normal tissue has been described: higher exposures of radiation lead to better responses; at sufficiently high doses RT sterilizes even “radioresistant” tumors. Likewise for normal tissues, where higher doses lead to greater damage, [32], treatment related toxicity is a major cause for the failure of radiotherapy. A potential solution to this insuperable problem is radiosensitization; therefore, despite the laundry list of previously failed radiosensitizers such as misonidazole, motexafin gadolinium (Xcytrin), Efaproxyn (efaproxiral or RSR-13) and bortozemib (Velcade), tirapazamine, RSR-13, eniposide, topotecan, paclitaxel, cisplatin and IUDR, tumor-targeted radiosensitization remains an attractive, if utopian, strategy to improve local control or cure rates.

However, the feasibility of radiosensitization as a therapeutic strategy ultimately depends on the optimization of the delicate balance between efficacy and normal tissue toxicity. The use of the quinone Mitomycin C, for example, despite a preferential toxicity for hypoxic tumor cells, is limited due to cumulative myelosuppression. [33] Likewise, tirapazamine, the first hypoxic cytotoxin to enter clinical trials [34], may prematurely ‘preactivate’ in tissues with a modest degree of hypoxia such as the retina and dermis (1.5 % oxygen) before reaching the true hypoxic core of the tumor (0.5 % oxygen), leading to relatively poor selectivity for neoplastic cells and a narrow therapeutic index.

Given its solid-tumor efficacy profile, minimal toxicity, hypoxic cell preference, unique mechanism of action and synergy with radiation, RRx-001 has the potential to fill this treatment vacuum in the therapeutic arsenal as a radiosensitizer.
